# Being Informed or Getting the Product? How the Coexistence of Scarcity Cues and Online Consumer Reviews Affects Online Purchase Decisions

**DOI:** 10.1007/s12599-022-00772-w

**Published:** 2022-09-02

**Authors:** Andrea Wrabel, Alexander Kupfer, Steffen Zimmermann

**Affiliations:** 1 Institute of Business Analytics, Ulm University, Helmholtzstr. 22, 89081 Ulm, Germany; 2 Department of Information Systems, Production and Logistics Management, and Digital Science Center (DiSC), University of Innsbruck, Universitaetsstr. 15, 6020 Innsbruck, Austria

**Keywords:** Electronic commerce platform, Scarcity cues, Online consumer reviews, Online purchase decision

## Abstract

Scarcity cues, which are increasingly implemented on e-commerce platforms, are known to impair cognitive processes and influence consumers’ decision-making by increasing perceived product value and purchase intention. Another feature present on e-commerce platforms are online consumer reviews (OCRs) which have become one of the most important information sources on e-commerce platforms in the last two decades. Nevertheless, little is known about how the presence of scarcity cues affects consumers’ processing of textual review information. Consequently, it is unclear whether OCRs can counteract the effects of scarcity or whether OCRs are neglected due to scarcity cues. To address this gap, this study examines the effects of limited-quantity scarcity cues on online purchase decisions when participants have the possibility to evaluate textual review information. The results of the experimental study indicate that scarcity lowers participants’ processing of textual review information. This in turn increases perceived product value and has considerable negative consequences for the final purchase decision if the scarcity cue is displayed next to a low-quality product. The study’s findings provide relevant insights and implications for e-commerce platforms and policymakers alike. In particular, it highlights that e-commerce platforms can easily (ab)use scarcity cues to reduce consumers’ processing of textual review information in order to increase the demand for low-quality products. Consequently, policymakers should be aware of this mechanism and consider potential countermeasures to protect consumers.

**Supplementary Information:**

The online version contains supplementary material available at 10.1007/s12599-022-00772-w.

## Introduction

Almost all e-commerce platforms provide online review systems, where consumers may share their experiences with a product. Such online consumer reviews (OCRs) typically consist of a numerical rating (e.g., star rating) and a textual review. Especially the textual reviews represent a valuable source “to mitigate the uncertainty about the quality of a product” (Kwark et al. 2014, p. 93) in online shopping situations where consumers do not have an on-hand experience with a product prior to purchasing it (Kwark et al. 2014; Manes and Tchetchik 2018). Thus, it is not surprising that about 82% of US adults read – at least sometimes – OCRs before buying a product (Smith and Anderson 2016) and that online review systems are considered one of the most important features when shopping online (Rowe and Kingstone 2018).

Another feature that can increasingly be observed on e-commerce platforms are scarcity cues (e.g., “Only 3 left in stock”). While such cues provide information about product availability, they also impair consumers’ underlying cognitive processes by increasing perceived product value (e.g., Amirpur and Benlian 2015; Wu and Lee 2016), purchase or booking intention (e.g., Teubner and Graul 2020; Wu and Lee 2016) and the likelihood of impulsive purchases (Guo et al. 2017; Wu et al. 2020). Further, there is growing evidence that websites display scarcity cues in an inaccurate and untruthful way (Mathur et al. 2019) which is particularly harmful for consumers if the scarcity cues are used for low-quality products because of the potential increase in both, perceived product value and purchase intention.

Little is known, however, whether scarcity cues also affect the evaluation of more diagnostic product information like OCRs that allow consumers to learn about the quality of a product. Although few studies examine the effect of scarcity cues in the presence of OCRs (Li et al. 2021; Park et al. 2017), they all focus on numerical ratings only. Hence, these studies neglect the information content of textual reviews. As textual reviews often include highly relevant information about consumers’ experiences with a product and its quality, it is important to understand how scarcity cues affect consumers’ processing of textual review information. This understanding is of particular relevance as textual review information might affect whether consumers purchase the product that fits best to their needs. We address this gap by answering the following research question:

**Research Question:** *How do scarcity cues affect online purchase decisions in the presence of textual review information?*

To answer this question, we conduct a between-subject online experiment where study participants have to choose between two products. By reading textual reviews, it is possible for the participants to identify that one product is of lower quality than the other product. While the control group receives the information that both products are available, we display a scarcity cue next to the low-quality product in the treatment group. This treatment variation allows us to examine whether scarcity affects consumers’ processing of textual review information and how this consequently affects online purchase decisions. In particular, we examine perceived product value, which we define as the relative difference between the willingness-to-pay for the high- and low-quality product, and decision accuracy, which we define as participants’ choice for the high-quality product.

To develop our hypotheses regarding the effects of scarcity in the presence of OCRs, we draw on commodity theory (Brock 1968) and on the competitive arousal model of decision-making (Ku et al. 2005). Commodity theory states that the value of a commodity is perceived as higher if it is scarce (Brock 1968). The competitive arousal model of decision-making developed by Ku et al. (2005) suggests that factors (scarcity in our case) triggering an aroused state can lead to an impaired decision-making process.

Based on our analysis of decisions from 615 participants of our incentive-compatible experiment, we obtain the following findings: First, the presence of a scarcity cue lowers the processing of textual review information. Second, scarcity affects perceived product value directly and indirectly via processed textual review information. In particular, the perceived value of the low-quality product (being the scarce product) increases relative to the perceived value of the high-quality product implying that participants perceive the values of both products less differently. Third, participants’ decision accuracy is indirectly decreased by scarcity via both, processed textual review information and perceived product value, if the low-quality product is indicated to be scarce. Fourth, we find that the effect of scarcity on the processing of textual review information disappears for participants who actively decide to see textual reviews and that the number of participants who actively decide to see textual reviews is significantly lower in the scarcity treatment. We conjecture that this finding could represent a self-selection mechanism caused by participants’ different state of arousal (due to scarcity). More specifically, those who actively decide to see textual reviews might be less aroused than those who decide to not process textual reviews and directly make their purchase decision.

Our study has important theoretical implications as we add to the understanding of scarcity cues in e-commerce platforms. By giving participants the possibility to evaluate the quality of a product by reading textual reviews, we observe that consumers’ cognitive processes in the presence of scarcity cannot only be explained by commodity theory. It further requires the competitive arousal model of decision-making to describe how consumers process textual review information. Interestingly, however, when participants actively decide to see textual reviews, the hypothesized effects derived from the competitive arousal model disappear. Consequently, we conjecture that this subsample of participants might be less aroused by scarcity. However, the effects of commodity theory persist as they are still affected by scarcity in terms of their perceived product value.

Our study also provides practical implications for e-commerce platforms and policymakers alike. As scarcity cues reduce the processing of essential information about a product’s experience attributes (e.g., in OCRs), they can easily be (ab)used by e-commerce platforms to increase the demand for low-quality products. Although such an (ab)use can also potentially harm e-commerce platforms due to lower consumer satisfaction and, in turn, higher product return rates, they are even more harmful for consumers. Thus, it is crucial that policymakers are aware of a potential misuse of scarcity cues and should take measures to protect consumers. In particular, policymakers could think about restricting the use of scarcity cues or incorporating countermeasures that reduce consumers’ arousal which would allow them to more carefully examine relevant product information.

## Related Literature

While scarcity has already been examined in classic offline shopping situations (e.g., Aggarwal et al. 2011; Parker and Lehmann 2011; Robinson et al. 2016; van Herpen et al. 2009; Worchel et al. 1975), the simplicity to introduce scarcity cues on e-commerce platforms has recently reignited interest in this research topic. For instance, current research on the effects of scarcity cues displayed on online platforms finds that their presence increases the perceived value of products and services (e.g., Amirpur and Benlian 2015; Teubner and Graul 2020; Wu and Lee 2016), leads to an increased purchase or booking intention (e.g., Song et al. 2019; Teubner and Graul 2020; Wu and Lee 2016) and also increases impulse purchases (Guo et al. 2017; Wu et al. 2020).

Although OCRs are known to be an important determinant for online purchase decisions as well (cf., e.g., Babić Rosario et al. 2016, 2020; Cheung and Thadani 2012; Floyd et al. 2014; Gutt et al. 2019 for comprehensive overviews), there is a lack of understanding whether scarcity cues impact consumers’ evaluation of OCRs and how this in turn affects purchase decisions on e-commerce platforms. We are only aware of two studies that examine the relationship between scarcity cues and numerical ratings. Park et al. (2017) experimentally study how different types of information cues (i.e., scarcity cues, popularity cues and numerical ratings) affect online hotel booking intention. While the authors observe a significant impact of popularity cues and numerical ratings on booking intention, they do neither find an effect of scarcity nor an interaction effect between scarcity cues and the numerical rating. In addition, a recent study by Li et al. (2021) examines the effect of scarcity cues on hotel booking intention during the COVID-19 pandemic and the moderating role of numerical ratings. In their online experiment, the authors observe that – during the COVID-19 pandemic – scarcity has a negative effect on booking intention, as it implies that a hotel is extensively booked and “social distancing” is hardly possible. However, if numerical ratings (reflected by a quality and a safety rating) are present as well, the negative effect of scarcity is reduced confirming the authors’ hypothesis on the moderating role of numerical ratings. However, these findings can neither be generalized for hotel booking in a non-pandemic environment nor for online shopping situations in general. Further, both of the studies described above focus on numerical ratings only and do not consider textual reviews which include, by definition, more extensive information about the quality of a product or a service.

Hence, no study has – to the best of our knowledge – yet examined how scarcity cues displayed on e-commerce platforms influence consumers’ processing of textual review information while making online purchase decisions, even though it is generally argued that scarcity influences rational decision-making by distorting information processing (Cialdini 2013). In this study, we address this research gap and apply an experimental approach that allows us to analyze whether the presence of a scarcity cue impairs consumers’ processing of textual review information and how this affects the subsequent purchase decision.

## Theoretical Background and Hypotheses Development

This section discusses the theoretical background on how scarcity affects consumers’ processing of textual review information, their perceived product value and their decision accuracy. As for some hypotheses (i.e., H2b and H3b) the direction of the hypothesized effects depends on product quality (i.e., textual review information) and as the (ab)use of scarcity cues for low-quality products is of higher relevance for consumers (e.g., mispurchase, lower customer satisfaction, more product returns, etc.), we will focus on the effects of scarcity for low-quality products in the following.^1^

### Effect of Scarcity on Processed Textual Review Information

Scarcity affects consumers’ information processing^2^ and decision-making by inducing a state of arousal (Cialdini 2013). In general, arousal represents a possible emotional response to environmental stimuli and can range from sleep to excitement over various intermediate states of drowsiness and alertness (Russell and Mehrabian 1977). Cialdini (2013) suggests that scarcity induces such a “brain clouding arousal” and thus, “our typical reaction to scarcity hinders our ability to think […] [and] cognitive processes are suppressed by our emotional reaction to scarcity pressures” (Cialdini 2013, pp. 255–256). Hence, the author concludes that scarcity impairs the careful examination of a situation. Further, as stated in the competitive arousal model of decision-making developed by Ku et al. (2005), factors that trigger an aroused state, like, e.g., “auction fever”, lead to an impaired decision-making process. Ku et al. (2005) further emphasize that competitive arousal can occur in various decision-making contexts and therefore suggest a broad applicability of this model for decisions under time pressure and competition. Based on the competitive arousal model of decision-making, we expect that the presence of scarcity impairs a rational decision-making process by inducing an aroused state. In this vein, Lewinsohn and Mano (1993) examine the relationship between arousal and decision-making and conclude that arousal induces less deliberation and less information processing and leads to fewer product-describing attributes being focused on.

On e-commerce platforms, the most relevant product-describing attributes are represented in OCRs as they provide previous consumers’ experiences with a product (Kwark et al. 2014). Consequently, we expect consumers’ processing of information given in textual reviews to be impaired by the aroused state induced by scarcity. In this context, we define processed textual review information as the amount of information consumers process from textual reviews (in form of product-describing attributes).^3^ Thus, consumers are expected to process less textual review information if a product is indicated to be scarce. Therefore, we state our first hypothesis as follows:

**H1:** Scarcity decreases consumers’ processed textual review information.

### Effects of Scarcity on Perceived Product Value

Considering the effect of scarcity on the perception of a product’s value, previous research finds that the perceived value of a product or service is higher, if it is scarce (e.g., Brock 1968; Worchel et al. 1975). This effect can be described by commodity theory (Brock 1968). The main principle of commodity theory refers to the statement that “any commodity will be valuated to the extent that it is unavailable” (Brock 1968)*.* In other words, according to commodity theory, scarcity leads to an increased value of a commodity (i.e., anything that is useful to its possessor and that can be transferred to another person). The applicability of commodity theory could also be confirmed for perceived product value by, e.g., Worchel et al. (1975) or van Herpen et al. (2009). Hence, we expect that the indication of scarcity has a positive direct effect on the perceived value of a product (i.e., the commodity in our case). Therefore, we state our next hypothesis as follows:

**H2a:** Scarcity directly increases the perceived value of a product.

Moreover, perceived product value is also influenced by OCRs. In this vein, several studies find that OCR valence affects product evaluation and purchase intention (Huang and Chen 2006; Lee et al. 2008; Tata et al. 2020; Ziegele and Weber 2015). As we hypothesize that scarcity decreases processed textual review information (cf., H1), we expect that consumers’ perceived product value is also less affected by OCRs. Importantly and as outlined above, the direction of this effect depends on product quality and we focus on the effects of scarcity for low-quality products. Textual reviews of low-quality products typically include a negative evaluation of product quality in form of negative textual review information. Consequently, as scarcity leads to less processing of textual review information, we expect that scarcity increases the perceived value of a low-quality product. In other words, we expect an indirect effect of scarcity on perceived product value with processed textual review information as mediating variable. Accordingly, we hypothesize:

**H2b:** Scarcity displayed next to a low-quality product indirectly increases the perceived product value via processed textual review information.

Overall, we expect scarcity displayed next to a low-quality product to have a positive direct effect on perceived product value (cf., H2a) as well as a positive indirect effect via processed textual review information (cf., H2b).

### Effects of Scarcity on Decision Accuracy

Decision accuracy (i.e., choosing the product that fits best to one’s needs) can be influenced by scarcity in three possible ways. First, decision accuracy can be influenced by the mediating effect of processed textual review information alone. It is well-known that consumers base their decisions on the choices and opinions of other individuals (e.g., Huang and Chen 2006) to reduce information asymmetries about a product (Manes and Tchetchik 2018; Park and Lee 2009) and that consumers show a higher purchase intention towards products that have a higher OCR valence (Huang and Chen 2006; Tata et al. 2020; Ziegele and Weber 2015). In other words, the evaluation of textual reviews helps consumers to make better purchase decisions. As we hypothesize that scarcity lowers consumers’ processing of textual review information (cf., H1), we expect that consumers cannot properly evaluate a product’s quality which has a negative consequence on decision accuracy. Therefore, we hypothesize:

**H3a:** Scarcity decreases decision accuracy indirectly via processed textual review information.

Second, as we hypothesize based on commodity theory that scarcity directly increases perceived product value (cf., H2a), we also expect the likelihood of purchasing the scarce product to increase. The direction of this effect again depends on product quality. Hence, for scarcity cues being displayed next to low-quality products, the perceived product value is expected to increase (cf., H2a) and the consumer is more likely to actually purchase the low-quality product which lowers decision accuracy. Hence, our next hypothesis reads as follows:

**H3b:** Scarcity displayed next to a low-quality product decreases decision accuracy indirectly via perceived product value.

Third, decision accuracy can also be affected by scarcity via a serial mediation over processed textual review information and perceived product value. As perceived product value is also indirectly affected via processed textual review information (cf., H2b), we expect this to have a consequence on decision accuracy as well: As hypothesized in H2b, we expect the perceived value of the low-quality product to increase due to less processed textual review information which in turn decreases decision accuracy (i.e., being more likely to purchase a low-quality product).^4^ Hence, we state our final hypothesis as follows:

**H3c:** Scarcity decreases decision accuracy indirectly via serial mediation through processed textual review information and perceived product value.

Overall, we expect scarcity displayed next to a low-quality product to indirectly affect decision accuracy in three possible ways: single mediation via processed textual review information (cf., H3a), single mediation via perceived product value (cf., H3b) and serial mediation via processed textual review information and perceived product value (cf., H3c).

**Fig. 1:**
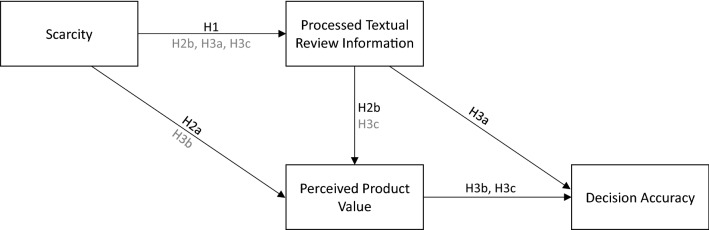
Research model

Figure 1 outlines our research model and refers to the respective hypotheses developed above.

## Research Methodology and Study Design

We apply an experimental approach to examine how scarcity influences the processing of textual review information and consumers’ online purchase decisions. An experimental investigation explicitly allows us to isolate the effects hypothesized above. This would be hardly possible by examining real-world data from an e-commerce platform as we cannot observe what products are displayed to the consumer, in which order they are presented and whether there are scarcity cues present or not. Further and even if we would be able to examine the processing of textual review information in the field, it seems nearly impossible to account for all other external factors that influence consumers.

### Scenario Description

**Fig. 2:**
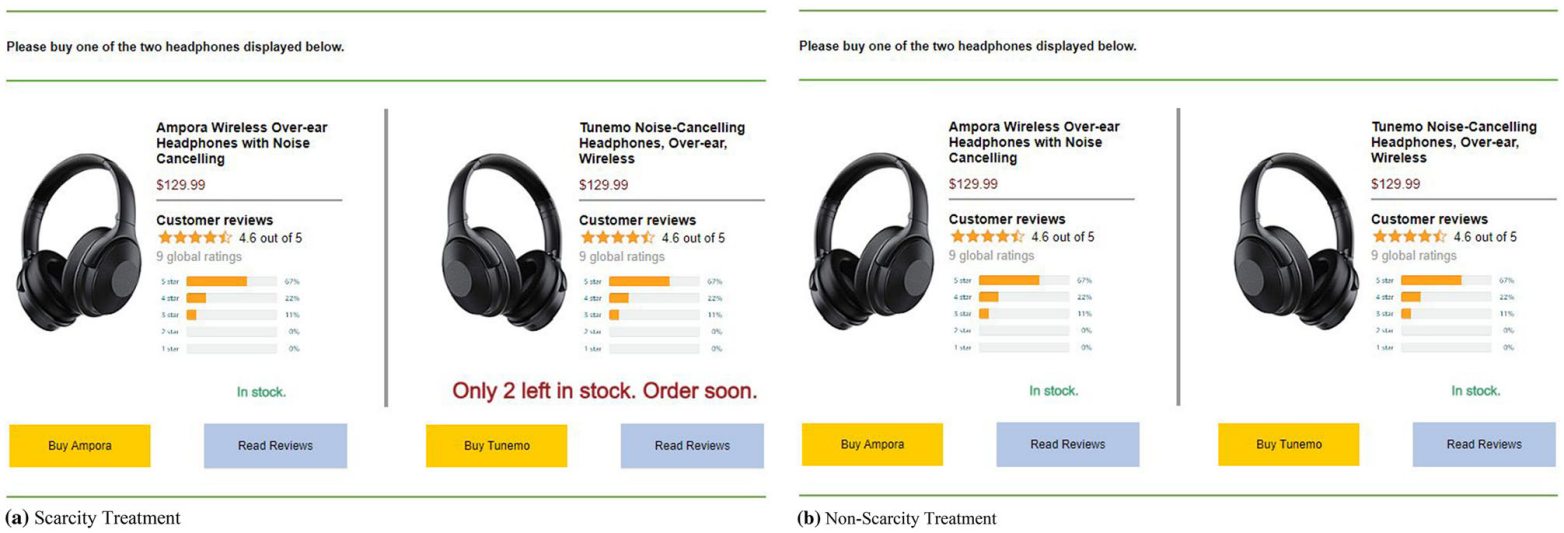
Purchase page in the experiment

In our scenario-based experiment, participants visit a fictive e-commerce platform and are asked to purchase a pair of noise cancelling headphones. On the purchase page of the e-commerce platform, two different headphones are offered. Both headphones have the same price (i.e., $129.99), offer the same features according to the title (i.e., over-ear, wireless and noise cancelling), look very similar (i.e., same photo only mirrored), have a brand-unrelated name (i.e., “Ampora” and “Tunemo”) and exhibit the same OCR metrics (i.e., number of reviews, average rating and rating distribution). The headphones, however, differ in terms of their quality which can only be assessed by reading the respective textual reviews. More specifically, the textual reviews indicate one product to be of high quality (i.e., “high-quality product” in the following) and the other product to be of low quality (i.e., “low-quality product” in the following). Textual reviews are not displayed in the first place but are accessible to all participants after clicking on a button “Read Reviews” which is next to the purchase button (cf., Fig. 2). Note that once a participant clicked on one of the two “Read Reviews” buttons, the textual reviews for both products are displayed. The participants’ main task is to choose one of the two headphones offered. For a more realistic and incentive-compatible setting, we informed participants that the payment depends on their decision during the experiment and that they receive an additional bonus payment if they make the “right” purchase decision (i.e., choosing the high-quality product). In this way, we create an incentive for participants to actually use all available information for their purchase decision in the experiment (cf., Sect. 4.5 for further details).

### Treatment Variation

Our experiment has a between-subjects design consisting of a non-scarcity and a scarcity treatment. In the non-scarcity treatment (control group), the information “In stock” is displayed for both headphones. In the scarcity treatment (treatment group), however, we replace “In stock” with a limited-quantity cue for the low-quality product. Figure 2 shows the purchase page for the scarcity treatment (Panel a) and the non-scarcity treatment (Panel b). As outlined before, we deliberately display the scarcity cue next to the low-quality product. The limited-quantity scarcity cue is implemented by displaying “Only $x$ left in stock. Order soon.” where $x$ is counting down from six to one as long as the participants view the purchase page. To avoid an order bias, we also randomize the position of the scarce product on the purchase page.

### Textual Review Creation and Content

As outlined above, the OCR metrics are the same for both headphones but the textual reviews (which are displayed after clicking the “Read Reviews” button) help to assess the quality of the headphones. To be specific, we display nine OCRs for each product with five OCRs being equivalent in their textual review content for both headphones. Four of the nine OCRs indicate that important product features are malfunctioning for the low-quality product (i.e., battery life, microphone, connectivity with multiple devices and quick charge function) while these product features are not criticized in the textual reviews for the high-quality product. For the wording of all textual reviews, we were inspired by actual textual reviews on headphones.

As we display the same OCR metrics and the same distribution of numerical ratings for both headphones, we also need to include low-rated textual review information for the high-quality product. We thereby focus, however, on very subjective side aspects that are not linked to the product quality itself (i.e., aversion to the color of the hard case and dislike of the amount of packaging material). To avoid that participants decide based on these aspects, similar information is also included in the OCRs for the low-quality product (i.e., favoring the color of the hard case and appreciating the amount of packaging material). Finally, as one could expect participants to pay most attention to the OCRs with the lowest numerical rating (i.e., 3-star rating), we display similar textual reviews for both headphones which, however, represent irrelevant information for the actual experimental task (i.e., headphones do not fit well on a child’s head). This prevents participants from instantly identifying the low-quality product. The OCRs consist of six 5-star reviews, two 4-star reviews and one 3-star review. Besides the textual review and the numerical (i.e., star) rating, each review also shows a fictional name of the reviewer. Table 1 below lists all OCRs for the high- and low-quality product. The rows 1) to 4) contain the textual review information that substantially differs in terms of quality. Row 5) shows the 3-star review with the irrelevant information for the experimental task. As before, we also randomize the position of the OCRs to avoid an order bias.

**Table 1:** List of OCRs

Attribute	High-quality product	Low-quality product
1) Battery life	David (★★★★★) Even after months, the battery life is amazingly long!	Susan (★★★★☆) Battery life was good at the beginning, but after a few months it decreases
2) Microphone	Christian (★★★★★) Amazing quality and the integrated microphone works really well!	Heather (★★★★☆) Good headphones, however I’ve sometimes issues with the microphone
3) Quick charge	Andrea (★★★★★) The quick charge mode really helps a lot and the required cable is already included	Kelly (★★★★★) I like the quick charge feature. Unfortunately, it requires an additional cable that is not included
4) Multi-device connection	Vanessa (★★★★★) Best headphones ever and it’s so easy to switch between different devices	Tyler (★★★★★) Good quality. However, it’s sometimes difficult to connect them with more than one device
5) Great head-phones	Jonas (★★★☆☆) Good headphones, but they do not fit on the small head of my 5 year old daughter	Jason (★★★☆☆) I like these headphones. Unfortunately they do not fit on my 6 year old son’s head
6) Active noise cancelling	Luisa (★★★★★) I must say that I really love the noise cancelling function and it is working perfectly	Ethan (★★★★★) The noise cancelling function is very useful and it is functioning pretty good
7) Sound quality	Benjamin (★★★★★) Of all headphones I ever owned, this model has the best sound quality!	Mike (★★★★★) I really like the sound of this model, even though my last headphones had a slightly clearer sound
8) Delivery	Karen (★★★★☆) I love these headphones! Fast delivery, but there was a lot of packaging material	Skylar (★★★★★) Good headphones! They were delivered really fast and well protected by the packaging material
9) Hardcase	Daniel (★★★★☆) Amazing sound! They come with a protective hard case but I do not like the color of the hard case	Scarlett (★★★★★) Good sound! I also like the hard case as it protects the headphones very well and it has a nice color

### Variables

#### Scarcity

Our independent variable represents the scarcity cue that is displayed next to the low-quality product in the scarcity treatment. In the non-scarcity treatment, “In Stock.” is displayed next to both products. Comparing the decisions between both treatments allows us to analyze the impact of scarcity on processed textual review information, perceived product value and decision accuracy.

#### Processed Textual Review Information

As stated in our hypotheses, we expect processed textual review information to be a mediating variable between scarcity and perceived product value as well as between scarcity and decision accuracy. We operationalize processed textual review information ($$ProcTextInfo$$) as the amount of information – in form of product-describing attributes – they process from textual reviews. The measurement of $$ProcTextInfo$$ is associated with previous research asking participants to recall product attributes (Lu et al. 2021; Pang and Qiu 2016). To obtain a quantifiable measure of the amount of information that was processed by participants, we asked participants to assign statements about product features (which were mentioned in the OCRs) to the corresponding headphones after their online purchase decision. In total, participants have to assign six statements of which two refer to the high-quality product, two refer to the low-quality product, one refers to both products and one to none of them. For each correctly assigned statement, $$ProcTextInfo$$ increases by one. Our experimental design also allows us to identify participants that actively decided to not process textual review information (i.e., those who did not click on the “Read Reviews” button). This is an important and substantial difference to the recall measures used by Lu et al. (2021) or Pang and Qiu (2016). Thus, $$ProcTextInfo$$ captures more than what participants can recall from textual reviews. To incorporate this aspect, we set $$ProcTextInfo$$ to zero if they do not click on the “Read Reviews” button. Consequently, $$ProcTextInfo$$ ranges from 0 to 7 and additionally differentiates between participants who decide to not process OCRs at all (i.e., $$ProcTextInfo=0)$$ and participants who see OCRs (and might process textual reviews in some way) but completely fail to assign the correct features (i.e., $$ProcTextInfo=1$$).

#### Perceived Product Value

One of our variables of interest and a potential mediator for the effects of scarcity on decision accuracy is perceived product value. To capture perceived product value, we asked participants to state the maximum amount they are willing to pay for each of the two headphones, independent of the given price. To avoid problems with the participant-specific, absolute level of the willingness-to-pay, we use the relative difference between the willingness-to-pay for both products. To be specific, we calculate for each participant the relative difference between the willingness-to-pay for the high-quality ($WT{P}_{hq}$) and low-quality ($WT{P}_{lq}$) product as ${\Delta }_{WTP}=(WT{P}_{hq}-WT{P}_{lq})/WT{P}_{lq}$.^5^

#### Decision Accuracy

As it is possible for participants to assess the quality of both products, they can identify which product is of low and high quality. In this vein, we define decision accuracy ($DecAcc$) as a binary variable being one if participants choose the high-quality product and zero if they choose the low-quality product.

#### Control Variables

We include several control variables in our analysis. First, we measure participants’ persuasion knowledge ($PersuaKnow$) using the items described in Bearden et al. (2001), as this might affect the participants’ response to the scarcity cue. Second, we ask for participants’ emotional perception during the decision-making process. This includes whether it was difficult to choose between the products ($ChoiceDiff$) and whether the decision-making process was stressful ($Stressful$). For both, persuasion knowledge and emotional perception, we used 5-point Likert scales ranging from 1 (strongly disagree) to 5 (strongly agree). Third, we ask participants about their familiarity with the topic. Thus, we ask whether they own, plan to buy or are not interested in noise cancelling headphones ($HeadphoneUser$), how often they shop online ($ShoppingExp$) and how often they read OCRs when shopping online ($ReviewReader$). Finally, we also ask for sociodemographic factors ($Age$, $Gender$, $Education$ and $Income$).

### Attention Check Questions and Incentive-Compatible Payment

We implement attention check questions to check whether participants carefully take part in the experiment and to ensure the quality of our data. Importantly, these questions are asked after the purchase decision, are very basic and can be answered without seeing the OCRs. For both treatments, we ask participants based on true/false answer options about the price of the headphones and whether the headphones have a noise cancelling feature. For the scarcity treatment, we additionally require that participants observe the scarcity cue and consequently ask whether one of the headphones was indicated to run out of stock (again with a true/false answer option).

As we want participants to make well-considered decisions, we implement the following incentive-compatible payout scheme: All participants receive a base payment of $0.10 for completing the experiment and receive additional $0.50 for correctly answering the attention check questions. We further include an additional bonus payment of $0.50 if the participants choose the high-quality product. Note that in this way, we create an incentive-compatible payout scheme (i.e., the better the participants’ decisions and answers, the higher their final payment). Thus, final payments range from $0.10 up to $1.10. Participants were aware of the bonus payments and that their payment depends on their own decisions during the experiment.

### Participants and Procedure

**Fig. 3:**
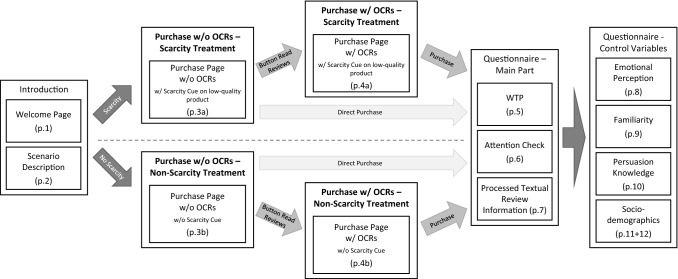
Experimental procedure

For our experiment, we recruited participants through Amazon Mechanical Turk (MTurk) and randomly assigned them to either the scarcity or the non-scarcity treatment. In total, 829 participants completed the survey. The experimental procedure is illustrated in Fig. 3 (see the Online Appendix for a complete outline of the experiment; available via 10.1007/s12599-022-00772-w).

On the welcome page of the experiment (i.e., page 1), we informed participants about the incentive-compatible payout scheme by highlighting that their payment depends on their decisions and answers to the attention check questions. To ensure that all participants were aware of our incentive-compatible payout scheme, they had to click on a button “*I understand that my payment is variable and depends on my attention and decisions*” to be forwarded to the scenario description.

On the scenario description page (i.e., page 2), participants were advised to imagine that they need new noise cancelling headphones. We further informed them that they will see a typical purchase page of an e-commerce platform similar to amazon.com on the next page, where they have to choose between two different headphones offered. After clicking on a button to ensure that they agree to have read and understood the instructions, participants were forwarded to the fictive purchase page (cf., Fig. 2).

On this purchase page (i.e., pages 3a and 3b), participants saw the fictive e-commerce website where they could either directly purchase the headphones or click on a button to be forwarded on a purchase page containing OCRs (i.e., pages 4a and 4b). Further, they had to decide which headphones to purchase by clicking on the purchase button. To ensure that participants do not accidentally click on the purchase button, we added a pop-up window where participants need to confirm their online purchase decision or could go back to the purchase page.

After their online purchase decision, we asked participants to state their willingness-to-pay for each product (i.e., page 5) and checked if they carefully attended the experiment by asking the attention check questions (i.e., page 6). On page 7, we measure processed textual review information by asking participants to assign statements about product features that were only mentioned in the textual reviews of the corresponding headphones. Then, participants were asked about their emotional perception during the decision-making process (i.e., page 8) and about their familiarity with noise cancelling headphones, online shopping and reading reviews (i.e., page 9). Finally, we asked participants about their persuasion knowledge (i.e., page 10) and sociodemographic information (i.e., pages 11 and 12).

### Implementation and Pre-testing

We implemented the experiment using the web-based survey software SoSci Survey. Prior to conducting the experiment, we performed two pre-tests with 100 participants each to examine if they can follow the experimental instructions and to eliminate potential issues and ambiguities (Reynolds and Diamantopoulos 1998). For consistency reasons, we also recruited the participants for the pre-tests via Amazon MTurk. Within the pre-tests, participants also had the possibility to give feedback on our experiment. Based on this feedback, we adapted several aspects of the experiment. First, we rephrased the willingness-to-pay question to ensure that participants understand the question correctly. Second, we adjusted the textual reviews in order to focus on objective quality features and reduced the total number of OCRs. Third, we simplified the wording of the scenario description and emphasized the fact that the participants’ payment depends on their answers to the attention check questions as well as on their own decisions.

## Analysis and Results

### Summary Statistics

Before analyzing the data, we only kept participants that answered all attention check questions correctly. 632 of the 829 recruited participants had done so. Next, we checked the dataset for outliers and deleted participants with values greater than the 99th percentile of the sample for ${\Delta }_{WTP}$ and for the time to complete the purchase decision, as well as participants whose ${\Delta }_{WTP}$ could not be calculated (17 observations were removed in total). Our final dataset includes 615 participants with 287 participants belonging to the scarcity treatment and 328 to the non-scarcity treatment. To get a first impression of the data, Table 2 presents the summary statistics for our main and control variables which are separated into the non-scarcity and scarcity treatment. The last column of Table 2 shows the difference in means between both treatments and whether this difference is statistically significant.

**Table 2:** Summary statistics

	Non-scarcity treatment (n = 328)				Scarcity treatment (n = 287)				Difference in means
	Mean	SD	Min	Max	Mean	SD	Min	Max	
**Main variables**									
$ProcTextInfo$	2.67	1.89	0	7	2.37	1.93	0	7	− 0.30**
${\Delta }_{WTP}$	21.9%	34.2%	− 50.0%	207.7%	13.8%	30.8%	− 42.9%	200.0%	− 8.1%***
$WT{P}_{hq}$	113.97	44.6	25	325	107.97	42.19	5	300	− 6.00**
$WT{P}_{lq}$	98.47	44.9	14	400	98.76	42.56	5	300	0.29
$DecAcc$	85.4%	35.4%	0	1	79.4%	40.5%	0	1	− 6.0%*
**Control variables**									
$PersuaKnow$	3.91	0.51	2.33	5	3.97	0.54	2.33	5	0.06
$ChoiceDiff$	3.28	1.12	1	5	3.36	1.04	1	5	0.08
$Stressful$	2.33	1.14	1	5	2.31	1.13	1	5	− 0.02
$HeadphoneUser$	1.79	0.85	1	3	1.91	0.89	1	3	0.12
$ReviewReader$	1.40	0.63	1	4	1.45	0.69	1	4	0.05
$ShoppingExp$	2.22	0.71	1	4	2.20	0.71	1	4	− 0.02
$Age$	3.55	1.20	2	6	3.54	1.23	1	6	− 0.01
$Gender$	1.62	0.50	1	3	1.64	0.48	1	2	0.02
$Education$	4.44	1.31	1	8	4.45	1.48	1	8	0.01
$Income$	4.40	2.13	1	8	4.10	2.20	1	8	− 0.30*

***p < 0.01; **p < 0.05; *p < 0.1. Statistical significance for differences in means is based on a one-sided t-test ($\mathrm{ProcTextInfo}$ and ${\Delta }_{\mathrm{WTP}}$), a Chi-square test ($\mathrm{DecAcc}$) and a Wilcoxon rank-sum test (all control variables). Note that a negative value for ${\Delta }_{\mathrm{WTP}}$ indicates that the value of the high-quality product is perceived as lower than the value of the low-quality product. For completeness, the raw values for ${\mathrm{WTP}}_{\mathrm{hq}}$ and ${\mathrm{WTP}}_{\mathrm{lq}}$, which are used to calculate ${\Delta }_{\mathrm{WTP}}$, are displayed as well

We observe that processed textual review information ($ProcTextInfo$) is significantly lower in the scarcity treatment indicating that scarcity negatively affects participants’ processing of textual review information during the online shopping process. Further, the relative difference in willingness-to-pay between the high- and low-quality product (${\Delta }_{WTP}$) is also significantly lower for the scarcity treatment: While participants in the scarcity treatment perceive the value of the high-quality product as 13.8% higher compared to the low-quality product, participants in the non-scarcity treatment perceive the value between the high-quality and low-quality product more differently (i.e., they value the high-quality product 21.9% higher than the low-quality product).^6^ Decision accuracy ($DecAcc$), indicating the percentage of participants who purchased the high-quality product, is also significantly lower in the scarcity treatment by 6 percentage points. As our control variables are all based on Likert scales, we apply a non-parametric Wilcoxon rank-sum test to examine the differences between the non-scarcity and the scarcity treatment. All variables except *Income* do not significantly differ from each other. We further investigate the difference in *Income* by applying a Chi-square test to analyze whether the frequencies for the answer options differ between treatments. The result of the Chi-square test suggests that the answer distributions between the non-scarcity and scarcity treatment do not significantly differ for *Income*. Consequently, we conclude that our sample randomization is appropriate.

### Effect of Scarcity on Processed Textual Review Information (Hypothesis 1)

While the univariate tests indicate a considerable influence of the scarcity cue on the main variables, we now turn to a multivariate regression analysis to test our hypotheses. We start with testing the effect of scarcity on processed textual review information by estimating the following ordinary least squares (OLS) model:

1 $$ProcTextInfo=\alpha +\beta ScarcityDum+\delta Controls+\epsilon ,$$

where $ProcTextInfo$ is the dependent variable. The independent variable of interest is $ScarcityDum$, which is a dummy variable being one for the scarcity treatment and zero for the non-scarcity treatment. $Controls$ is a vector that includes all control variables outlined above. $\epsilon$ represents the remaining error term. Robust standard errors were used.

As shown in Table 3 below, we observe a negative and statistically significant coefficient ($\beta =-0.324, p<0.05$) of $ScarcityDum$. Thus, participants in the scarcity treatment have a significantly lower amount of processed textual review information. This supports our Hypothesis 1: the presence of a scarcity cue in our experiment lowers the participants’ processing of textual review information.

**Table 3:** Effect of scarcity on processed textual review information (OLS regression)

	Effect
$ScarcityDum$ → $ProcTextInfo$ (H1)	− 0.324** (0.150)

***p < 0.01; **p < 0.05; *p < 0.1. Robust standard errors are used and shown in parentheses. All control variables are included

### Effects of Scarcity on Perceived Product Value (Hypotheses 2a and 2b)

To examine the impact of scarcity on perceived product value, we first analyze the hypothesized direct effect (cf., H2a) by estimating the following OLS model:

2 $${\Delta }_{WTP}=\alpha +\beta ScarcityDum+\gamma ProcTextInfo+\delta Controls+\epsilon ,$$

where we use ${\Delta }_{WTP}$, being the relative difference in willingness-to-pay between the high- and low-quality product, as our operationalization for perceived product value. As independent variables, we include $ScarcityDum$ as well as $ProcTextInfo$. As before, we also include all control variables ($Controls$) and use robust standard errors.

The results for the OLS model are shown in the first row of Table 4. We observe a significant direct effect $\left(\beta =-0.070, p<0.01\right)$ of scarcity on ${\Delta }_{WTP}$. The negative sign of the coefficient implies that participants perceive the values of both products less differently in the scarcity treatment (compared to the non-scarcity treatment). In other words, this implies that the perceived value of the low-quality product (being the scarce product) increases relative to the perceived value of the high-quality product. Thus, we find support for Hypothesis 2a.

**Table 4:** Effects of scarcity on perceived product value

	Effect	SE	LLCI	ULCI
$ScarcityDum$ → ${\Delta }_{WTP}$ (H2a)	**−** **0.070**	0.024	− 0.118	− 0.022
$ScarcityDum$ → $ProcTextInfo$ → ${\Delta }_{WTP}$ (H2b)	**−** **0.022**	0.011	− 0.044	− 0.003

Robust standard errors are used. Standard errors (SE) and 95% confidence intervals (95% CI; LLCI/ULCI = Lower/Upper Limit of Confidence Interval) for the indirect effect are based on 5,000 bootstrapping resamples. Mediation analysis is based on the PROCESS macro Model 4 (Hayes, 2017). All control variables are included. Effects in bold font indicate statistical significance at the 5%-level

To test whether processed textual review information mediates the effect of scarcity on perceived product value (cf., H2b), we apply a mediation analysis using the PROCESS macro (Model 4) for SPSS (Hayes 2017). We thereby assess the statistical significance of indirect effects via a bootstrapping procedure as, according to Hayes (2017), the bootstrapping confidence interval tends to have higher power compared to the traditional test by Sobel (1982). Hence, we base standard errors and 95% confidence intervals for the indirect effects on 5,000 bias-corrected bootstrapping resamples. As for the direct effects, we include all control variables.

The results of the mediation analysis are shown in the second row of Table 4. In particular, we observe a significant indirect effect $\left(\beta =-0.022, p<0.05\right)$ of scarcity on ${\Delta }_{WTP}$ through processed textual review information. As before, the negative coefficient indicates that the value of both products is perceived less differently in the scarcity treatment. This again implies that the perceived value of the low-quality product (being the scarce product) increases relative to the perceived value of the high-quality product. Thus, we also find support for Hypothesis 2b.

By comparing the coefficients between the direct and indirect effect, we observe that the indirect effect accounts for approx. 24% of the total effect of scarcity on perceived product value (cf., Table 4). Hence, we observe a partial mediation for the effect of scarcity on perceived product value through processed textual review information.

### Effects of Scarcity on Decision Accuracy (Hypotheses 3a, 3b and 3c)

Finally, we analyze the effects of scarcity on decision accuracy. Prior to conducting the mediation analysis to test the hypothesized indirect effects via processed textual review information and perceived product value (cf., H3a–H3c), we examine whether there exists a potential (though not hypothesized) direct effect of scarcity. Thus, we estimate the following logistic regression model.

3 $$DecAcc=\alpha +\beta ScarcityDum+\gamma ProcTextInfo+\zeta {\Delta }_{WTP}+\delta Controls+\epsilon ,$$

where $DecAcc$ is a binary variable being one if a participant purchased the high-quality product and zero otherwise. To test for a direct effect of scarcity while accounting for potential indirect effects, we include $ScarcityDum$, $ProcTextInfo$ as well as ${\Delta }_{WTP}$ as independent variables. Again, $Controls$ represents the vector that includes all control variables outlined above and robust standard errors are used. The results are shown in the first row of Table 5 and we can confirm that there exists no significant direct effect of scarcity on decision accuracy.

**Table 5:** Effects of scarcity on decision accuracy

	Effect	SE	LLCI	ULCI
$ScarcityDum$ → $DecAcc$ (*not hypothesized*)	− 0.133	0.275	− 0.672	0.406
$ScarcityDum$ → $ProcTextInfo$ → $DecAcc$ (H3a)	**−** **0.174**	0.097	− 0.400	− 0.017
$ScarcityDum$ → ${\Delta }_{WTP}$ → $DecAcc$ (H3b)	**−** **0.775**	0.390	− 1.762	− 0.235
$ScarcityDum$ → $ProcTextInfo$ → ${\Delta }_{WTP}$ → $DecAcc$ (H3c)	**−** **0.249**	0.154	− 0.636	− 0.025

Notes for mediation analysis: robust standard errors are used. Standard errors (SE) and 95% confidence intervals (95% CI; LLCI/ULCI = Lower/Upper Limit of Confidence Interval) for the indirect effect are based on 5,000 bootstrapping resamples. Mediation analysis is based on the PROCESS macro Model 6 (Hayes, 2017). Effects are represented in log-odds metrics because of the binary dependent variable ($\mathrm{DecAcc}$). All control variables are included. Effects in bold font indicate statistical significance at the 5%-level

However, we hypothesize potential indirect effects of scarcity on decision accuracy in H3a (i.e., $ScarcityDum$ → $ProcTextInfo$ → $DecAcc$), H3b (i.e., $ScarcityDum$ → ${\Delta }_{WTP}$ → $DecAcc$) and H3c (i.e., $ScarcityDum$ → $ProcTextInfo$ → ${\Delta }_{WTP}$  → $DecAcc$), respectively. To test these hypotheses, we apply a serial multiple mediation analysis using the PROCESS macro (Model 6) for SPSS (Hayes 2017). Similar to the mediation analysis above, standard errors and 95% confidence intervals for the indirect effects are based on 5,000 bias-corrected bootstrapping resamples and we again include all control variables.

Results for the serial multiple mediation analysis are also shown in Table 5. The results indicate – in line with Hypotheses 3a, 3b and 3c – statistically significant indirect effects of scarcity on decision accuracy. More specifically, scarcity indirectly lowers decision accuracy via processed textual review information, via perceived product value and via the serial mediation of processed textual review information and perceived product value. As we do not observe a direct effect, the results suggest a full mediation of the effect of scarcity on decision accuracy.

So far, our findings can be summarized as follows: First, we find that scarcity lowers consumers’ processing of textual review information. Second, scarcity significantly affects the perceived product value directly as well as indirectly via the lowered amount of processed textual review information. Third, scarcity indirectly leads to less accurate purchase decisions via mediation through perceived product value, processed textual review information as well as serial mediation including both of them. As participants had to click on the “Read Reviews” button to see textual reviews, the next section focuses on those who actively made this decision to see OCRs.

### Clicker Subsample Analysis

To begin with, we examine how scarcity affects the decision to click on the “Read Reviews” button prior to making the purchase decision. In this context, it is important to recall that the experiment has an incentive-compatible payout scheme (i.e., better purchase decision increases participants’ payout) which ensures that participants have an incentive to use all information provided to make a good decision during the experiment. Further, we added a pop-up window where participants needed to confirm their online purchase decision asking them whether they are sure to proceed with the purchase. This pop-up window also eliminates the possibility that participants accidentally purchased the product.

Remarkably, while we observe that 82.4% of the participants in the non-scarcity treatment decided to see OCRs, only 74.6% of the participants did so in the scarcity treatment. This implies, in turn, that 17.6% of the participants in the non-scarcity treatment and 25.4% in the scarcity treatment directly purchased the product and decided to not process OCRs. To examine the statistical significance of this difference, we estimate the following logistic regression model:

4 $$ClickButton=\alpha +\beta\, ScarcityDum+\delta\, Controls+\epsilon ,$$

where $ClickButton$ represents a binary variable being one if participants clicked on the “Read Reviews” button prior to purchasing the headphones and zero otherwise. As independent variables, we include $ScarcityDum$ and all control variables. The coefficient of $ScarcityDum$ is negative and statistically significant ($\beta =-0.434, p<0.05$). Translating the coefficient to the ratio of the odds, it implies that the odds for the scarcity treatment to click on the “Read Reviews” button are about 35% lower than the odds for the non-scarcity treatment. Thus, scarcity significantly lowers the participants’ likelihood to click on the “Read Reviews” button and in turn, as a direct consequence, increases the likelihood to directly purchase the headphones.

In a next step, we focus on those participants who actively decided to see OCRs by clicking on the “Read Reviews” button. To begin with, Table 6 presents the summary statistics of the subsample for our main variables. While we still observe the main variables to be lower in the scarcity treatment, only the perceived product value exhibits a statistically significant difference based on a one-sided t-test.

**Table 6:** Summary statistics for the clicker subsample

	Non-scarcity treatment (n = 270)				Scarcity treatment (n = 214)				Difference in means
	Mean	SD	Min	Max	Mean	SD	Min	Max	
${\Delta }_{WTP}$	25.7%	35.4%	− 50.0%	207.7%	18.5%	33.2%	− 34.0%	200.0%	− 7.1%**
$DecAcc$	91.1%	28.5%	0	1	87.9%	32.7%	0	1	− 3.3%
$ProcTextInfo$	3.24	1.57	1	7	3.18	1.55	1	7	− 0.06

***p < 0.01; **p < 0.05; *p < 0.1. Statistical significance for differences in means is based on a one-sided t-test ($\mathrm{ProcTextInfo}$ and ${\Delta }_{\mathrm{WTP}}$) and a Chi-square test ($\mathrm{DecAcc}$). Note that a negative value for ${\Delta }_{\mathrm{WTP}}$ indicates that the value of the high-quality product is perceived as lower than the value of the low-quality product

Although these univariate tests already indicate that scarcity is less influential for this subsample of participants, we perform the same multivariate analysis as above for this subsample as well. As in the main analysis, we again examine (i) the effect of scarcity on processed textual review information, (ii) the effect of scarcity on perceived product value as well as (iii) the effect of scarcity on decision accuracy for the subsample, respectively. We also include all control variables, use robust standard errors and simulate 5,000 bootstrap samples for the indirect effects of the mediation analyses.

The results are shown in Table 7. First, we no longer observe a significant effect of scarcity on processed textual review information. Second, perceived product value is only directly affected by scarcity (i.e., H2a) and the indirect effect via processed textual review information has vanished. Third, decision accuracy is as well only impacted by the single mediation via perceived product value (i.e., H3b). Hence, although scarcity does not distort participants’ processing of textual review information anymore, they are still affected by scarcity in terms of perceived product value which also negatively impacts their decision accuracy.

**Table 7:** Subsample analysis

Panel A: Effect of scarcity on processed textual review information	Effect
$ScarcityDum$ → $ProcTextInfo$ (H1)	− 0.118 (0.137)

Panel B: Effect of scarcity on perceived product value	Effect	SE	LLCI	ULCI
$ScarcityDum$ → ${\Delta }_{WTP}$ (H2a)	**−** **0.078**	0.029	− 0.135	− 0.020
$ScarcityDum$ → $ProcTextInfo$ → ${\Delta }_{WTP}$ (H2b)	− 0.008	0.009	− 0.027	0.011

Panel C: Effect of scarcity on decision accuracy	Effect	SE	LLCI	ULCI
$ScarcityDum$ → $DecAcc$ *(not hypothesized)*	− 0.258	0.391	− 1.023	0.508
$ScarcityDum$ → $ProcTextInfo$ → $DecAcc$ (H3a)	− 0.034	0.058	− 0.180	0.054
$ScarcityDum$ → ${\Delta }_{WTP}$ → $DecAcc$ (H3b)	**−** **1.229**	2.323	− 3.349	− 0.345
$ScarcityDum$ → $ProcTextInfo$ → ${\Delta }_{WTP}$ → $DecAcc$ (H3c)	− 0.132	0.405	− 0.620	0.214

Panel A: ***p < 0.01; **p < 0.05; *p < 0.1. Robust standard errors are used and shown in parentheses. All control variables are included

Panel B and C: Robust standard errors are used. Standard errors (SE) and 95% confidence intervals (95% CI; LLCI/ULCI = Lower/Upper Limit of Confidence Interval) for the indirect effect are based on 5,000 bootstrapping resamples. Mediation analyses are based on the PROCESS macro Model 4 for Panel B and Model 6 for Panel C (Hayes, 2017). All control variables are included. Effects in bold font indicate statistical significance at the 5%-level

## Discussion

### Contribution

This study contributes to existing research on the effects of scarcity on online purchase decisions. By examining the effect of scarcity cues in the presence of OCRs, we combine two important streams of IS literature. On the one hand, research exists that examines the effect of OCRs on online purchase decisions and finds that OCRs can qualitatively improve consumer decisions by learning about the quality of a product (Kwark et al. 2014; Manes and Tchetchik 2018). On the other hand, some studies analyze the effect of scarcity cues on online purchase decisions and observe that scarcity cues can distort consumers’ online purchase behavior (e.g., Guo et al. 2017; Wu et al. 2020; Wu and Lee 2016). Combining these streams of research allows us to analyze how scarcity cues influence consumers’ processing of textual review information and – as a consequence – their purchase decisions.

In our experiment, we observe a substantial impact of scarcity on participants’ decision accuracy via processed textual review information and perceived product value, confirming all of our hypotheses (cf., Table 8 Column “Full Sample”). In particular, we observe the following findings for the presence of a scarcity cue on a low-quality product: Scarcity lowers consumers’ processing of textual review information (H1). It further affects perceived product value by decreasing the difference between the willingness-to-pay for the high- and low-quality product directly (H2a) and indirectly via processed textual review information (H2b). Finally, we find that scarcity indirectly reduces decision accuracy via processed textual review information (H3a), via perceived product value (H3b) and serially via processed textual review information and perceived product value (H3c). Hence, displaying a scarcity cue next to a low-quality product results in fewer participants making the “right” purchase decision (i.e., choosing the high-quality product) even though they could have easily assessed both products’ quality.

**Table 8:** Summary of main findings

	Full sample	Clicker subsample
$ScarcityDum$ → $ProcTextInfo$ (H1)	✓	–
$ScarcityDum$ → ${\Delta }_{WTP}$ (H2a)	✓	✓
$ScarcityDum$ → $ProcTextInfo$ → ${\Delta }_{WTP}$ (H2b)	✓	–
$ScarcityDum$ → $ProcTextInfo$ → $DecAcc$ (H3a)	✓	–
$ScarcityDum$ → ${\Delta }_{WTP}$ → $DecAcc$ (H3b)	✓	✓
$ScarcityDum$ → $ProcTextInfo$ → ${\Delta }_{WTP}$ → $DecAcc$ (H3c)	✓	–

We acknowledge that these findings are considerably driven by participants that decide to not process textual review information at all. When further examining the subsample of participants who actively decided to see textual reviews, we only find support for Hypotheses 2a and 3b (cf., Table 8 Column “Clicker Subsample”). Hence, we conjecture that those participants clicking on the “Read Reviews” button might be less aroused than those who directly make their purchase decision without seeing textual review information. Nonetheless, their perceived product value is still affected by scarcity which also impacts their decision accuracy.

We further find that more than one quarter of the participants in the scarcity treatment decides to not process OCRs at all (and to buy the product directly), even though their payout was incentive-compatible and therefore dependent on their decisions. In the non-scarcity treatment, on the contrary, only approx. 18% of the participants decided to not process OCRs and to directly buy the product. Hence, a scarcity cue significantly decreases the propensity for the active decision to see textual reviews. Consequently, our findings in the subsample analysis could represent a self-selection mechanism caused by participants’ different state of arousal (due to scarcity). In particular, those who actively decide to see textual reviews might be less aroused than those who directly make their purchase decision without processing textual review information.

### Theoretical Implications

This study adds to the understanding of scarcity cues in combination with additional, product-related information. Guided by Brock’s (1968) commodity theory, we observe – in line with existing research – that scarcity cues directly affect perceived product value. Moreover, our study provides the first evidence that scarcity cues also impact consumers’ processing of textual review information and consequently perceived product value and purchase decisions which can be explained by Ku et al.’s (2005) competitive arousal model of decision-making. Hence, we add to the understanding of the effects of scarcity cues in e-commerce by highlighting that consumers’ cognitive processes cannot solely be explained by commodity theory. As our findings suggest that the effect of scarcity is substantially higher when taking arousal into account, it is likely that existing studies underestimate the relevance of scarcity cues as they neglect the evaluation of additional and more diagnostic information like OCRs.

Although we expected that the hypothesized effects from the competitive arousal model also exist for the subsample of clickers, we observe that scarcity does not influence processed textual review information in this subsample. In other words, the effect of scarcity derived from the competitive arousal model disappears when consumers actively decide to see textual reviews. Hence, only the direct effect on perceived product value (i.e., H2a) and the resulting mediation on decision accuracy (i.e., H3a) remain for the subsample of clickers indicating that clickers are not aroused by scarcity and only commodity theory drives the effects for this subsample.

### Practical Implications

Our study also provides practical implications for e-commerce platforms and policymakers alike. We find that scarcity cues do affect consumers’ purchase decisions. While in offline purchase settings consumers can easily evaluate the presence of scarcity by simply looking at a store’s shelves, this is not possible on e-commerce platforms. Thus, e-commerce platforms could easily (ab)use scarcity cues to reduce the processing of textual review information and consequently to increase the demand for low-quality products. However, such an (ab)use of scarcity cues can be a double-edged sword for e-commerce platforms and should be used with caution as it might also lead to lower consumer satisfaction and, in turn, to higher product return rates. In addition, e-commerce platforms could even make profits out of the arousal that is induced by displaying scarcity cues. For instance, platforms could offer consumers the opportunity to reserve products for a fixed amount of money and a fixed amount of time in the online shopping basket. Aroused consumers might be willing to pay for this reservation option as it allows them to calmly evaluate product quality by reading textual reviews and make the “right” purchase decision.

On the other hand, policymakers should be aware that a potential misuse of scarcity cues harms consumers and should carefully think about potential platform restrictions and countermeasures to protect consumers. One policy measure to counteract this potential misuse could be to only allow (limited-quantity) scarcity cues if they are true. If policymakers track or allow consumers to report the misuse of scarcity cues, they could sanction the misusing platforms. Having ensured by such sanctions that only true scarcity cues are present on e-commerce platforms, consumers could decide based on true information whether they first want to read OCRs (and take the risk that the product might be sold out) or whether they want to own the product in any case as they might urgently need it (and take the risk of lower quality). Referring to the reservation option described above, a policymaker could also simply require e-commerce platforms to implement such a feature to reserve products in the shopping basket, but prohibit the platform from charging money for this reservation. With this policy measure, consumers can be certain that items in their shopping basket will not be sold out in the next, e.g., 15 min, and they could use this time to evaluate the quality of a product with less time pressure. The reality, however, is currently quite different as some e-commerce platforms in fact caution consumers that their products are not reserved in the shopping basket, thus putting even more pressure on the consumer.^7^

### Limitations and Future Research

Our study has certain limitations, which, however, might serve as starting points for future research.

First, our experiment created an artificial online shopping situation using a scenario-based approach. While we ensured that the experimental design is incentive-compatible, participants still had to immerse themselves in a situation in which they need to buy new headphones. Hence, our results can only indicate how scarcity cues affect consumers’ online purchase decisions in the presence of OCRs in the real world. To address this issue, future research could transfer our setting to a real online shopping situation by cooperating with an e-commerce platform and displaying scarcity cues to one out of two groups of consumers (A/B testing).

Second, we only examined the effects of scarcity for displaying a scarcity cue next to the low-quality product. For high-quality products, we expect that Hypotheses 1, 2a, 3a, and 3c still hold while the direction of the hypothesized effects in Hypotheses 2b and 3b changes. In our incentive-compatible choice experiment, we were not able to test these expectations, because displaying the scarcity cue next to the high-quality product does not allow us to distinguish whether participants choose the product because of the higher quality or because of the scarcity cue. Thus, to support our expectations from above, future research should examine the effect of scarcity for high-quality products with a modified experimental design.

Third, we investigated the effect of one specific type of scarcity cue (i.e., limited-quantity). While this cue represents the most prevalent one, it would nonetheless be worthwhile to investigate how other scarcity cues (e.g., limited-time cues or popularity cues) influence consumers’ processing of textual review information. Hence, future research could conduct a higher factorial study design to analyze how additional scarcity cues and their interaction with each other impact consumers’ processing of textual review information.

Fourth, the online shopping situation in our experiment focused on a specific product (i.e., headphones). Future research could still examine other products (e.g., low-priced vs. higher-priced products) or services. Furthermore, future research studying the effects of scarcity on the processing of textual review information and online purchase decisions could also vary the type of e-commerce platform. As OCRs are of particular importance in markets with high asymmetric information (e.g., consumer-to-consumer sharing markets), it might be interesting to examine whether scarcity cues have a similar effect on sharing platforms.

Fifth, we observe a self-selection of participants into two subsamples with one subsample that actively decides to see textual reviews and one subsample that directly buys the product without seeing OCRs. While we conjecture that those participants clicking on the “Read Reviews” button might be less aroused by scarcity than those who directly make their purchase decision, this claim needs to be examined. Hence, future research could perform a similar experiment and concentrate on potential differences between participants by examining personal characteristics (e.g., risk aversion, personality traits). In the same vein, future research could also qualitatively examine participants’ survey responses after being exposed to a scarcity cue. This would improve our understanding of whether consumers perceive the urgency from a scarcity cue as more important than the risk of purchasing a low-quality product.

Sixth, we measure processed textual review information by examining whether participants actually decide to see textual reviews and, if so, how many statements about product features (which were mentioned in the OCRs) they can assign correctly. Even though this measure is similar to the recall of product attributes/reviews used in previous literature (Lu et al. 2021; Pang and Qiu 2016), future research could use other measures or apply other methods to capture participants’ processing of textual review information. In this context, eye tracking could, for instance, represent a promising method to identify information processing strategies of participants (Ryan et al. 2018).

Future research could further examine the effectiveness of our proposed policy measures to reduce the effect of scarcity on consumers’ processing of textual review information (e.g., reserving products). Hence, future research could examine different measures that are expected to reduce the arousal which is induced by scarcity cues and analyze their effects on consumers’ purchase decisions. Moreover, future research could also consider the visual salience of the scarcity cue and OCRs to find further aspects that might strengthen or weaken the effect of scarcity on consumers’ online purchase decisions. In this vein, it might also be interesting to immediately show OCRs on the purchase page without requiring participants to click on a button. In addition, there might also be differences due to the device consumers use for online shopping (i.e., smartphone vs. computer) which could be investigated.

## Supplementary Information

Below is the link to the electronic supplementary material.

Supplementary file 1 (PDF 936 KB)

## Footnotes

1 Nonetheless, the hypotheses could analogously be developed for high-quality products being scarce. It is, however, not our intention and scope to manipulate the quality of the scarce product in our study.

2 Research on information processing in the context of online reviews and electronic word-of-mouth without any scarcity cues is often based on dual-process theory (e.g., Cheung and Thadani 2012; Gupta and Harris 2010; Lee et al. 2008) considering the elaboration likelihood model (Petty and Cacioppo 1986) or the heuristic-systematic model (Chaiken 1980). Both models are consistent with our developed hypotheses if consumers are motivated and willing to invest cognitive effort. In a real online shopping situation, it is obvious, that consumers are motivated to find the product that fits best to their needs. In our experiment, we ensure this condition with an incentive-compatible payout scheme.

3 Note that processed textual review information differs from recall of product attributes/reviews used in previous literature (Lu et al. 2021; Pang and Qiu 2016). For more details on this difference please refer to Sect. 4.4 where the variables used in our experiment are defined.

4 Importantly, this effect on decision accuracy is independent of product quality. The perceived product value is impaired by scarcity due to less processed textual review information (i.e., increased for low-quality products, decreased for high-quality products) and therefore leads to a reduced decision accuracy in both cases.

5 Importantly, this implies that we would expect the perceived value of the low-quality product to be “closer” to the high-quality product in the scarcity treatment. In other words, we would expect to see a reduced ${\Delta }_{WTP}$ as the product values of the two headphones are perceived as more similar.

6 Although the raw values for $WT{P}_{hq}$ and $WT{P}_{lq}$ displayed in Table 2 are counterintuitive at first glance, it is important to note that it is not valid to compare the raw $WTPs$ across participants for the following reason: As we asked participants to state their $WTPs$ for both headphones simultaneously (see Online Appendix), it is likely that the first $WTP$ is an anchor for the second $WTP$. In other words, when a participant starts with entering the $WTP$ for, e.g., the low-quality headphones (and knowing that they are of low quality), the subsequent $WTP$ for the high-quality headphones will be higher but the first $WTP$ is used as a reference point. Therefore, we focus on the relative difference between both $WTPs$ as this is independent of reference points and, furthermore, ignores participant-specific price levels (e.g., some participants might value this type of headphones generally higher/lower).

7 On the German Online Shopping Platform AboutYou.de for instance, the corresponding information reads as follows: “Items in the basket are not reserved”.
