# Supplementary material for: Being Informed or Getting the Product? How the Coexistence of Scarcity Cues and Online Consumer Reviews Affects Online Purchase Decisions
Source: Bus Inf Syst Eng. 2022 Sep 2;64(5):575–92. doi: 10.1007/s12599-022-00772-w (PMC9438392; doi:10.1007/s12599-022-00772-w)
Supplement: Supplementary file 1 — (PDF 936 KB) [file 12599_2022_772_MOESM1_ESM.pdf]

Being Informed or Getting the Product?

How the Coexistence of Scarcity Cues and Online Consumer Reviews Affects Online Purchase Decision

Andrea Wrabel, Alexander Kupfer, Steffen Zimmermann

Business & Information Systems Engineering (2022)

Appendix (available online via http://link.springer.com)

Online Appendix

The following online appendix includes a complete outline of the online experiment. The page numbers refer to Figure 3 shown in the manuscript.

Page 1: Welcome Page

Welcome to the experiment and thank you for participating!

On the next pages, you will see a fictive online shopping situation and you will be asked to buy a product. Please read all information provided during the experiment carefully and try to immerse yourself into the situation as good as possible.

Please be aware that your payment depends on your decisions and answers during the experiment. For participating in the survey, you will automatically receive $0.1 as payment.

In addition, this experiment also includes control mechanisms to check your attention. Depending on your decisions during the experiment and answers to the attention checks, you will receive an additional bonus of up to $1.0.

All data collected will be held anonymously and will be used for academic research purposes only.

I understand that my payment is variable and depends on my attention and decisions

Page 2: Scenario Description

The online shopping situation:

Imagine that your employer has instructed you to work more from home due to the pandemic. Your work especially involves a lot of online meetings and tasks where you have to concentrate a lot. Thus, in order not to be disturbed by a noisy environment, you decided to buy new noise-cancelling headphones.

On the next page, you will see a typical online shopping website similar to amazon.com. On this page, you have to **choose between two different headphones offered.**

Please note: **There are four buttons on the next page and all of them are interactive**.

To start the experiment, please confirm that you have read and understood the instructions.

I have read and understood the instructions

Page 3: Purchase Page

Please note that we also varied the position of the scarce product (right/left) on the purchase page which will not be shown in detail. Further, we implemented the following pop-up window for each treatment to ensure that participants do not accidently click on the purchase buttons. Thus, participants can either confirm their purchase decision or go back to the page.

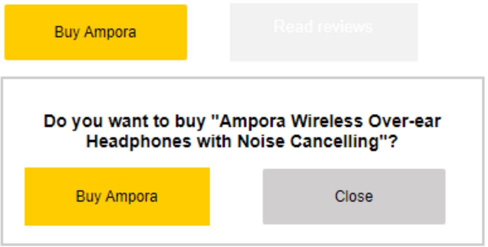

Page 3a: Scarcity-Treatment

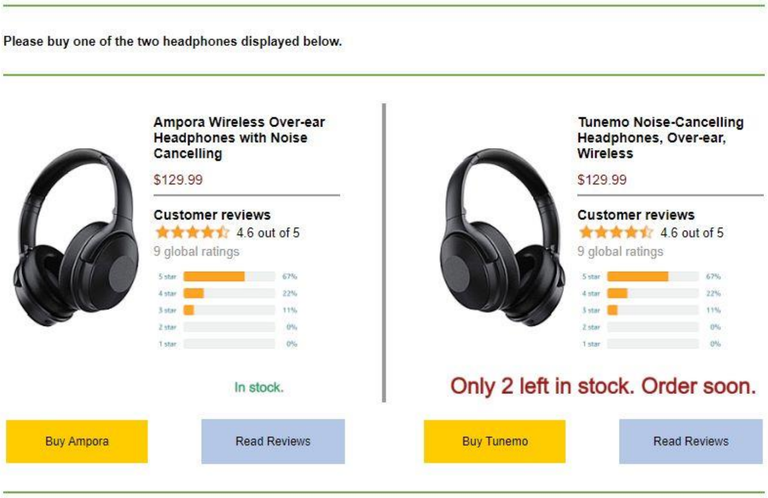

Note: If participants click on "Read Reviews", the site expands as follows:

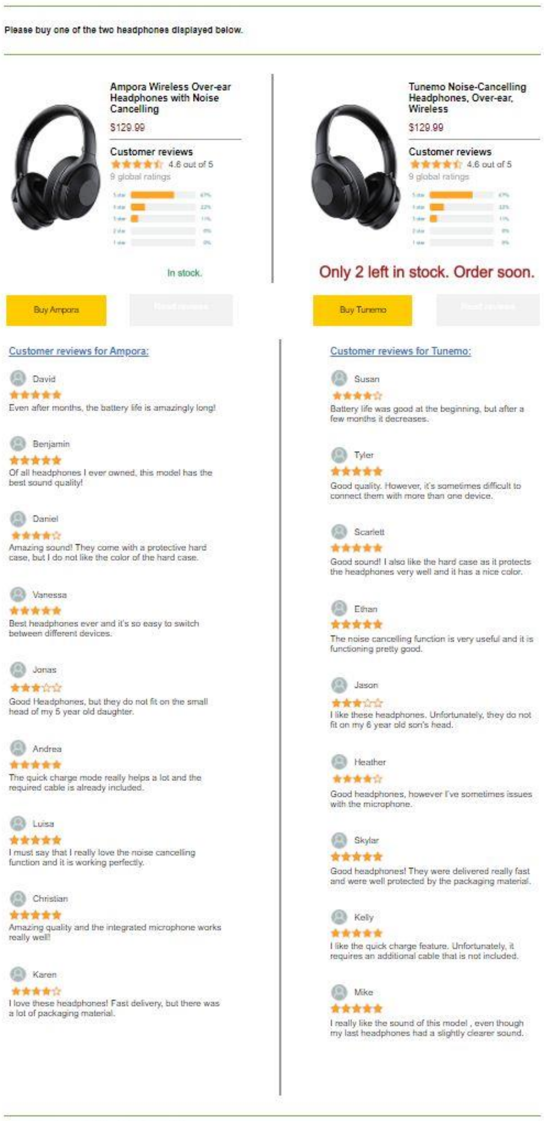

Page 3b: Non-Scarcity Treatment

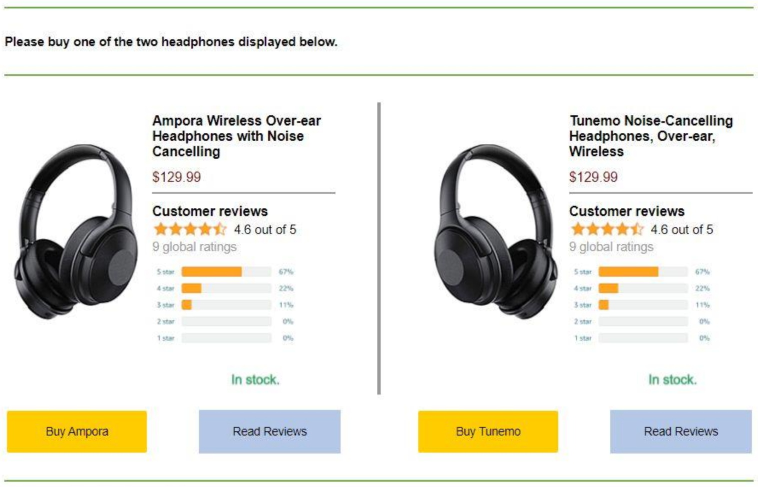

Note: If participants click on “Read Reviews”, the site expands as follows:

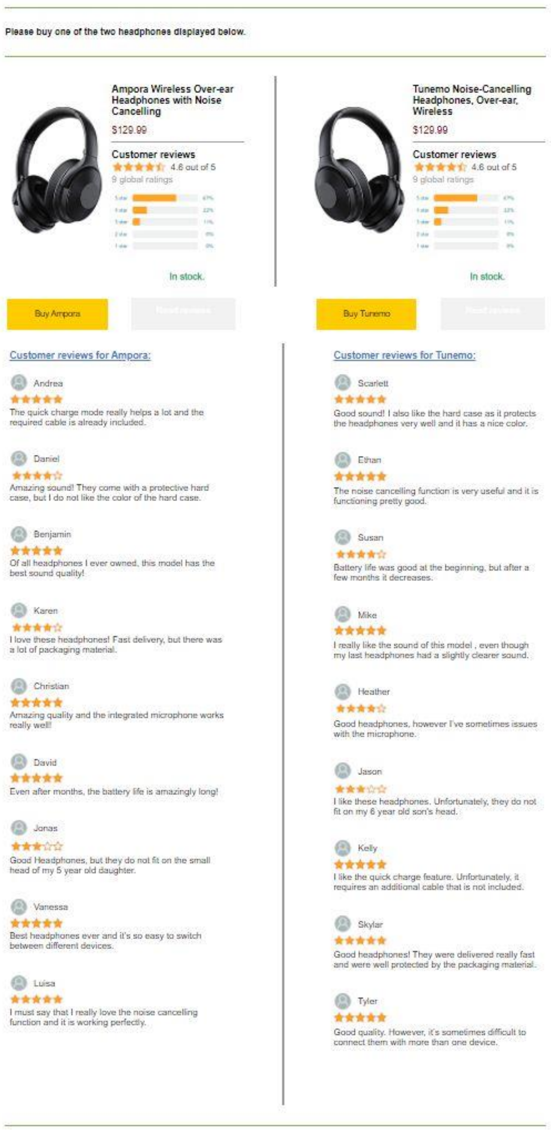

Bridging Page

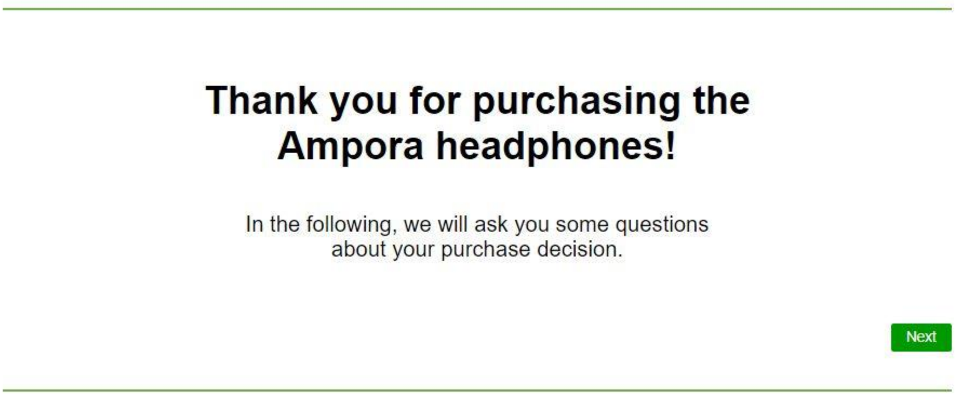

Note: If participants choose the other headphones (i.e., Tunemo), “Ampora” is replaced by “Tunemo”.

Page 5: Willingness-to-pay

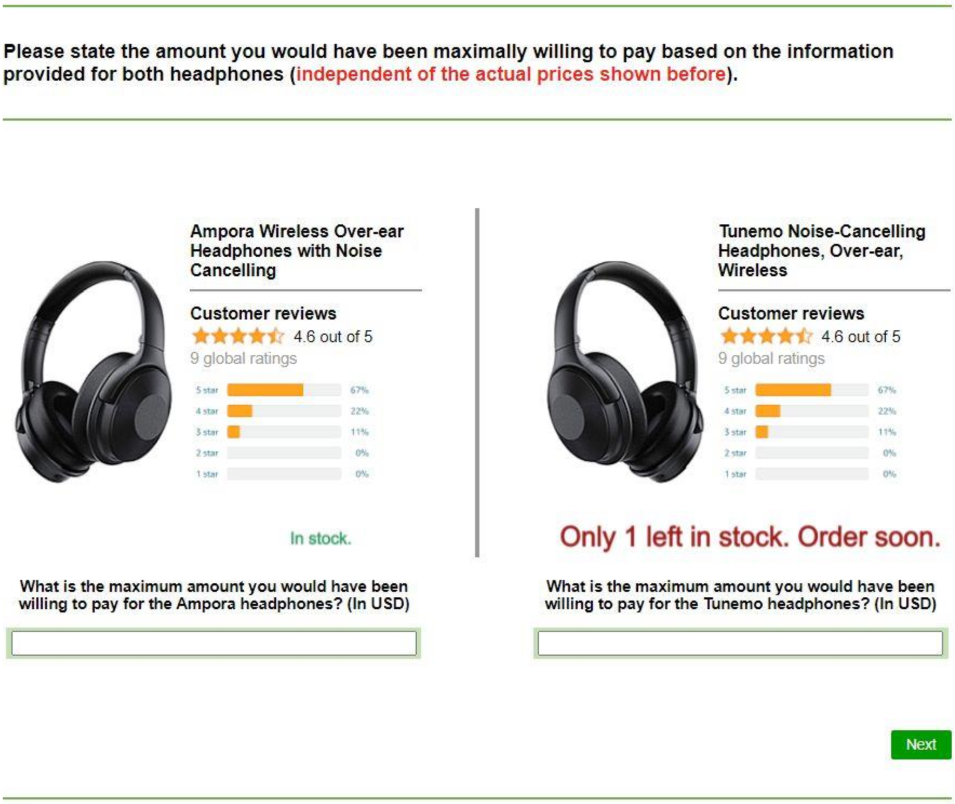

Note: This Figure is shown in the scarcity treatment only. In the non-scarcity treatment, “In Stock” is displayed instead of the scarcity cue.

Page 6: Attention Check Questions

In the following, we want to check your attention during the purchase process. Hence, please state whether the following statements are true or false.

	True	False	I cannot remember
The price of the headphones you chose was $99.99	○	○	○
One of the headphones displayed before was indicated to run out of stock.	○	○	○
Both pairs of headphones have a noise cancelling function.	○	○	○

Next

Page 7: Processed Textual Review Information

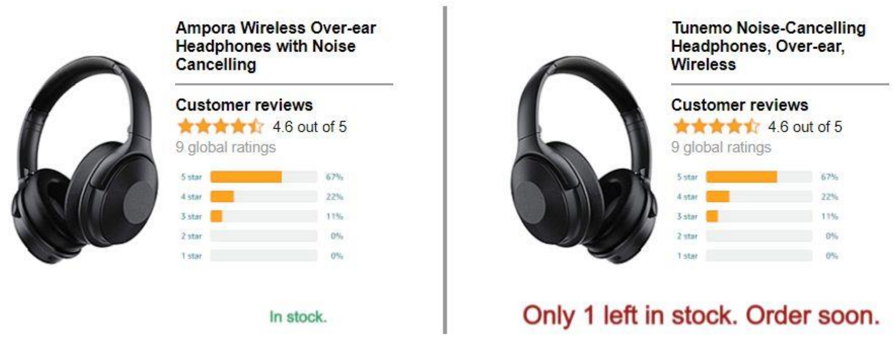

Please try to assign the attributes listed below to the appropriate product.

	Ampora	Tunemo	Ampora and Tunemo	Attribute not mentioned	I cannot remember
The microphone works properly.	○	○	○	○	○
Battery life is decreasing.	○	○	○	○	○
The headphones come with a hard case.	○	○	○	○	○
Connectability with multiple devices is difficult.	○	○	○	○	○
Easily usable quick charge function.	○	○	○	○	○
Outside voice call quality is good.	○	○	○	○	○

Next

Note: This Figure is shown in the scarcity treatment only. In the non-scarcity treatment, “In Stock” is displayed instead of the scarcity cue.

Page 8: Emotional Perception

How did you feel during the decision process?

	Strongly disagree	Disagree	Neutral	Agree	Strongly Agree
It was easy for me to choose between the two headphones.	○	○	○	○	○
I felt stressed during the purchase process.	○	○	○	○	○
I was afraid that my preferred headphones were going to be sold out.	○	○	○	○	○

Next

Page 9: Familiarity

All of the remaining questions do not refer to the experiment situation anymore. Instead they refer to you and your personal experience. Please answer them accordingly.

What is your experience with noise-cancelling headphones?

- ○ I own noise-cancelling headphones.
- ○ I am planning to buy noise-cancelling headphones.
- ○ I am not interested in noise-cancelling headphones.

How often do you shop online?

- ○ Daily
- ○ Weekly
- ○ Monthly
- ○ Rarely

How often do you read online reviews when deciding on making an online purchase?

- ○ (Almost) Every Time
- ○ In more than 50% of my online purchases
- ○ In less than 50% of my online purchases
- ○ Never

Next

Page 10: Persuasion Knowledge

How strongly do you agree with the following statements?

	Strongly disagree	Disagree	Neutral	Agree	Strongly Agree
I know when an offer is "too good to be true".	○	○	○	○	○
I can tell when an offer has strings attached.	○	○	○	○	○
I have no trouble understanding the bargaining tactics used by salespersons.	○	○	○	○	○
I know when a marketer is pressuring me to buy.	○	○	○	○	○
I can see through sales gimmicks used to get consumers to buy.	○	○	○	○	○
I can separate fact from fantasy in advertising.	○	○	○	○	○

Next

Page 11: Sociodemographics 1

What is your gender?

○ Male
○ Female
○ Other

What is your age?

○ 19 years or younger
○ 20 to 29 years
○ 30 to 39 years
○ 40 to 49 years
○ 50 to 59 years
○ 60 years or older

Which is the country, you are currently living in?

Next

Page 12: Sociodemographics 2

What is the highest educational qualification you have?

- ○ Less than high school diploma
- ○ High school diploma or GED
- ○ Some college, but no degree
- ○ Associates degree
- ○ Bachelor's degree
- ○ Master's degree
- ○ Professional degree
- ○ Doctorate or PhD

What is your total annual pretax income?

- ○ Less than $10,000
- ○ $10,000 - $25,000
- ○ $25,001 - $40,000
- ○ $40,001 - $55,000
- ○ $55,001 - $70,000
- ○ $70,001 - $85,000
- ○ $85,001 - $100,000
- ○ Above $100,000

Next
